# Quadriceps tendon autograft is becoming increasingly popular in revision ACL reconstruction

**DOI:** 10.1007/s00167-021-06478-y

**Published:** 2021-02-16

**Authors:** Philipp W. Winkler, Thiago Vivacqua, Stephan Thomassen, Lisa Lovse, Bryson P. Lesniak, Alan M. J. Getgood, Volker Musahl

**Affiliations:** 1 Department of Orthopaedic Surgery, UPMC Freddie Fu Sports Medicine Center, University of Pittsburgh, 3200 S. Water St., Pittsburgh, PA 15203 USA; 2 Department for Orthopaedic Sports Medicine, Klinikum rechts der Isar, Technical University of Munich, Ismaninger Str. 22, 81675 Munich, Germany; 3 Department of Orthopedic Surgery, Fowler Kennedy Sport Medicine Clinic, University of Western Ontario, 3M Centre, 1151 Richmond Street, London, ON N6A 3K7 Canada

**Keywords:** ACL, Anterior cruciate ligament, Revision, Lateral extra-articular tenodesis, Quadriceps tendon, Allograft

## Abstract

**Purpose:**

To evaluate trends in revision anterior cruciate ligament reconstruction (ACL-R), with emphasis on intra-articular findings, grafts, and concurrent procedures. It was hypothesized that revision ACL-Rs over time show a trend toward increased complexity with increased use of autografts over allografts.

**Methods:**

This was a two-center retrospective study including patients undergoing revision ACL-R between 2010 and 2020. Demographic and surgical data including intra-articular findings and concurrent procedures were collected and compared for the time periods 2010–2014 and 2015–2020. All collected variables were compared between three pre-defined age groups (< 20 years, 20–30 years, > 30 years), right and left knees, and males and females. A time series analysis was performed to assess trends in revision ACL-R.

**Results:**

This study included 260 patients with a mean age of 26.2 ± 9.4 years at the time of the most recent revision ACL-R, representing the first, second, third, and fourth revision ACL-R for 214 (82%), 35 (14%), 10 (4%), and 1 (< 1%) patients, respectively. Patients age > 30 years showed a significantly longer mean time from primary ACL-R to most recent revision ACL-R (11.1 years), compared to patients age < 20 years (2.2 years, *p* < 0.001) and age 20–30 years (5.5 years, *p* < 0.05). Quadriceps tendon autograft was used significantly more often in 2015–2020 compared to 2010–2014 (49% vs. 18%, *p* < 0.001). A high rate of concurrently performed procedures including meniscal repairs (45%), lateral extra-articular tenodesis (LET; 31%), osteotomies (13%), and meniscal allograft transplantations (11%) was shown. Concurrent LET was associated with intact cartilage and severely abnormal preoperative knee laxity and showed a statistically significant and linear increase over time (*p* < 0.05). Intact cartilage (41%, *p* < 0.05), concurrent medial meniscal repairs (39%, *p* < 0.05), and LET (35%, non-significant) were most frequently observed in patients aged < 20 years.

**Conclusion:**

Quadriceps tendon autograft and concurrent LET are becoming increasingly popular in revision ACL-R. Intact cartilage and severely abnormal preoperative knee laxity represent indications for LET in revision ACL-R. The high rate of concurrent procedures observed demonstrates the high surgical demands of revision ACL-R.

**Level of evidence:**

Level III.

## Introduction

Failure rates of up to 10% and 30% after primary and revision anterior cruciate ligament reconstruction (ACL-R), respectively, highlight the clinical relevance of revision ACL-R in daily clinical practice [12, 24, 37, 42, 44, 49, 57–59]. Accordingly, revision ACL-R has been extensively studied in recent years. In particular, the prospective longitudinal Multicenter ACL Revision Study (MARS) provided high-quality data with a high level of evidence for revision ACL-R and identified numerous independent predictors for the outcomes of revision ACL-R [5–8, 23–36, 39, 52]. However, patient enrollment in the MARS cohort ended in 2011 and based on the findings of the MARS the standard of care in revision ACL-R may have changed.

Knee ligament registries represent another valuable source which provide insights into outcomes, failure rates, and descriptive data of revision ACL-R [22, 23]. Despite large sample sizes and generalizability, prospectively collected data from national registries are subject to numerous limitations, including multiple surgeons and surgical techniques, misclassifications, and insufficient knowledge of confounding factors [54]. Consequently, it seems to be difficult to identify changes in practice patterns in revision ACL-R and the driving causes based on register studies alone.

Therefore, the purpose of this study was to evaluate trends in revision ACL-R based on two surgical sites, with emphasis on intra-articular findings, grafts, and concurrent procedures. It was hypothesized that revision ACL-Rs over time show a trend toward increased complexity in concurrent procedures with increased use of autografts over allografts.

## Materials and methods

Approval for this study was obtained by the institutional review boards of the University of Pittsburgh (No.: STUDY20050226) and the University of Western Ontario (No.: 101533). Given the retrospective design of this study, the need for written informed consent of the included patients was waived.

**Fig. 1:**
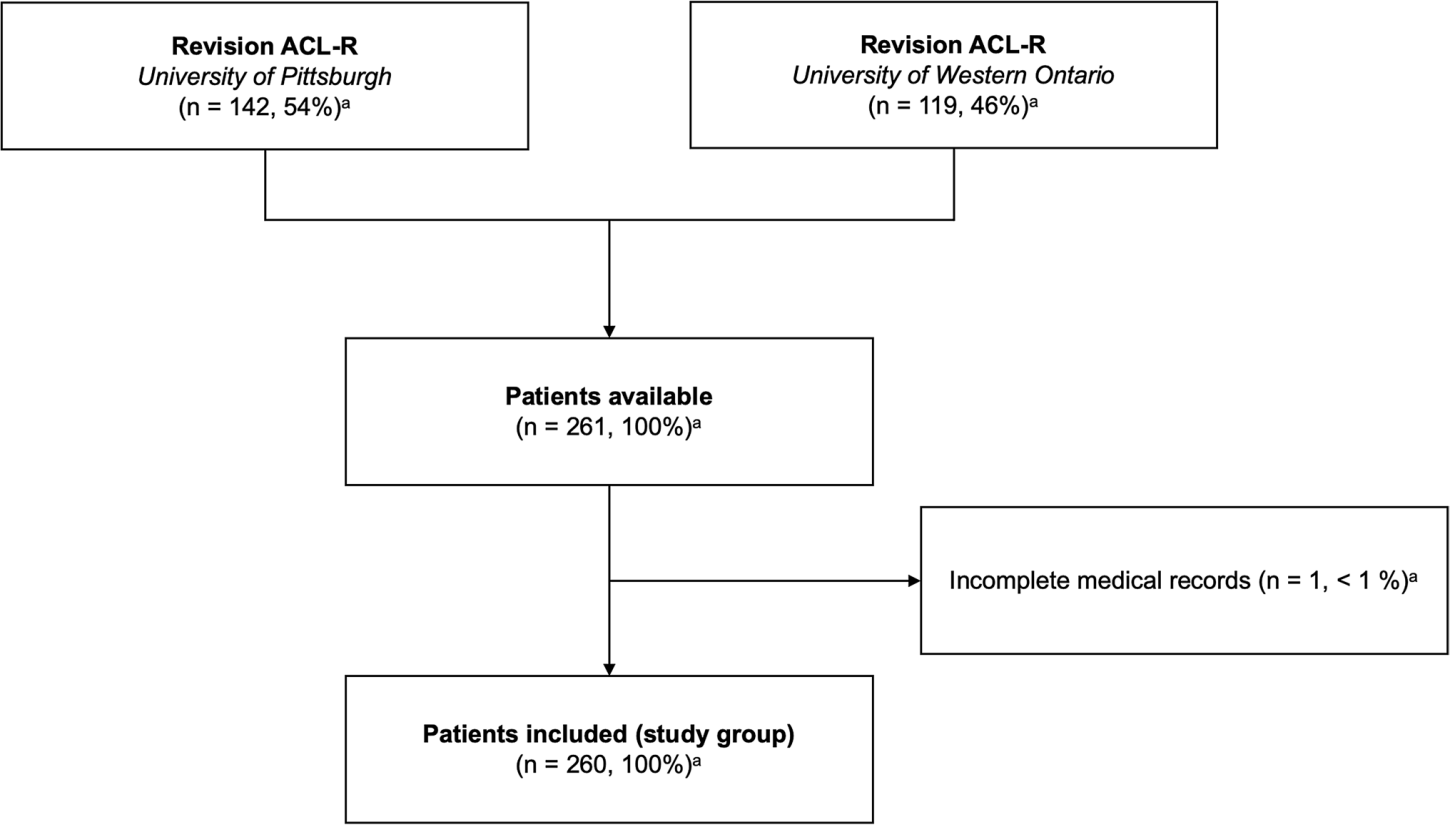
Flowchart of patient recruitment. ^a^Percentage of screened population undergoing revision ACL-R. *ACL-R* anterior cruciate ligament reconstruction

Patients undergoing revision ACL-R between 2010 and 2020 performed by fellowship-trained knee surgeons at the University of Pittsburgh (VM, BPL) and the University of Western Ontario (AMJG) were screened for eligibility for this retrospective two-center study (Fig. 1). Patients with inflammatory arthritis, previous proximal tibia or distal femur fractures, and incomplete medical records were excluded from the study. Included patients were assigned to one of three pre-defined groups based on the age at the time of the most recent revision ACL-R: < 20 years, 20–30 years, > 30 years. Two time periods, 2010–2014 and 2015–2020, were defined to determine changes in practice patterns over time.

### Revision ACL-R and concurrent procedures

Included patients underwent single-stage or two-stage anatomic single-bundle transportal revision ACL-R by one of the participating fellowship-trained knee surgeons (VM, BPL, AMJG). Graft choice for revision ACL-R was determined by the operating surgeon based on patient history (i.e., prior graft choice(s)), and preoperative (i.e., examination under anesthesia) and intraoperative (i.e., notch size, prior tunnel placement) findings. Grafts used included: ipsilateral and contralateral hamstring tendon, quadriceps tendon, and bone-patellar tendon-bone autografts, and fresh-frozen allografts. Concurrent procedures were categorized as follows: repair or reconstruction of the medial collateral ligament (MCL), lateral collateral ligament (LCL), or posterior cruciate ligament (PCL), meniscus surgery, cartilage surgery, osteotomy, and lateral extra-articular tenodesis (LET). Meniscus surgery was categorized as meniscal repair, partial meniscectomy, and meniscal allograft transplantation (MAT). Cartilage surgery included microfracture, osteochondral autograft and allograft transplantation, matrix-induced autologous chondrocyte implantation, and application of particulated juvenile articular cartilage [10]. The osteotomy types performed included: medial open wedge, medial closed wedge, lateral closed wedge, and anterior closed wedge high tibial osteotomy and medial closed wedge distal femoral osteotomy.

### Demographic and surgical data

A review of medical records was performed by one observer at each center (PWW, TV) between March and October 2020 to collect demographic and surgical data. Demographic data collected included sex, affected knee, body-mass-index (BMI), age at the time of most recent revision ACL-R, and the prevalence of contralateral anterior cruciate ligament (ACL) injuries. Surgical data from the most recent revision ACL-R collected included staging (single-stage vs. two-stage revision ACL-R), grade of preoperative Lachman and pivot-shift tests during examination under anesthesia, intra-articular findings, graft type, graft diameter, femoral and tibial graft fixation technique, and concurrently performed intra- and extra-articular surgical procedures. Furthermore, the number of revision ACL-R performed was documented. Preoperative Lachman and pivot-shift tests were graded as normal, nearly normal, abnormal, and severely abnormal according to the International Knee Documentation Committee (IKDC) Knee Examination Form. Cartilage conditions were evaluated according to the International Cartilage Repair Society (ICRS) grading system and were classified as intact cartilage (ICRS grade 0), low-grade lesion (ICRS grade 1 and grade 2), and high-grade lesion (ICRS grade 3 and grade 4). Meniscal lesions observed were categorized by location (medial, lateral) and type (horizontal, longitudinal, radial, ramp, complex, root, status post meniscal repair, status post partial meniscectomy). Four techniques of femoral graft fixation (suspensory, interference screw, hybrid, over-the-top) and three techniques of tibial graft fixation (suspensory, interference screw, hybrid) were defined. Hybrid graft fixation represents a combination of suspensory and interference screw graft fixation.

### Radiographic data

The medial and lateral posterior tibial slope (PTS), the anterior–posterior lateral femoral condyle length, and the lateral femoral condyle depth were measured on strict lateral radiographs, as previously described [41, 45]. Based on the lateral femoral condyle length and depth, the lateral femoral condyle ratio (LFCR) was calculated [45]. To avoid measurement inaccuracies, lateral radiographs with > 6 mm posterior femoral condyle overlap were excluded for the measurement of the medial and lateral PTS, and the lateral femoral condyle length and depth [45].

All measurements were performed by one observer at each center (PWW, TV). Inter- and intra-rater reliability of measurements between and within the two observers was evaluated based on 10 randomly selected patients. Intraclass correlation coefficients (ICC) were calculated and revealed good to excellent intra-rater (ICC 0.895–0.987) and moderate to excellent inter-rater (ICC 0.725–0.978) reliability of measurements.

### Statistical analysis

Categorical variables were expressed as number of patients and percentage of the corresponding group. Continuous variables were reported as mean ± standard deviation and range. Group comparisons with two independent variables (male vs. female; right vs. left; 2010–2014 vs. 2015–2020) were performed by the Chi-squared test for categorical variables and by the unpaired *t* test or Mann–Whitney *U* test for continuous variables. Group comparisons for the three pre-defined age groups (< 20 years vs. 20–30 years vs. > 30 years) were performed by the Chi-squared test (followed by a post hoc test with Bonferroni corrected *p* values) for categorical variables and by a one-way analysis of variance or the Kruskal–Wallis test (followed by post hoc testing) for continuous variables. To assess the trend of concurrently performed procedures over the 10-year period, a time series analysis (i.e., linear regression model) was performed. The percentage of combined revision ACL-R (ACL-R + meniscus surgery, ACL-R + LET, ACL-R + osteotomy) among all revision ACL-Rs performed annually represented the dependent variables, while the corresponding year represented the independent variable [2]. Given the descriptive design of this study and that all patients available were included, an a priori sample size calculation was not conducted. SPSS software version 26.0 (IBM-SPSS, New York, USA) was used for statistical analysis. The level of significance was defined as *p* < 0.05.

## Results

Out of 261 patients screened for eligibility, 1 (< 1%) patient had to be excluded due to incomplete medical records (Fig. 1). Thus, 260 patients with a mean age of 26.2 ± 9.4 years (range 13–58 years) at the time of the most recent revision ACL-R, were included in this study. The mean time from primary ACL-R to the most recent revision ACL-R was 6.1 ± 6.0 years (range 0.2–40.0 years) for the total study group. The most recent revision ACL-R represented a single-stage procedure in 223 (86%) patients and was the first, second, third, and fourth revision ACL-R for 214 (82%), 35 (14%), 10 (4%), and 1 (< 1%) patients, respectively. Concomitant MCL, PCL, and LCL injuries were observed in 13 (5%), 5 (2%), and 4 (2%) patients, respectively.

### Sex and laterality

Statistically significantly more MCL injuries were observed in males compared to females (8% vs. 1%, *p* < 0.05). Females were found to have statistically significantly more contralateral ACL injuries (18% vs. 8%, *p* < 0.05) and a higher LFCR (0.66 vs. 0.64, *p* < 0.001) compared to males. All other variables showed no statistically significant difference between males and females. No statistically significant differences were found between right and left knees for all variables analyzed.

### Age groups

#### Demographic, surgical, and radiographic data

A detailed overview is shown in Table 1. The mean time from primary ACL-R to the most recent revision ACL-R was 2.2 ± 1.6 years, 5.5 ± 3.5 years, and 11.1 ± 8.6 years for patients age < 20 years, 20–30 years, and > 30 years, respectively, representing a statistically significant difference between all three pre-defined age groups (< 20 years vs. 20–30 years, *p* < 0.001; < 20 years vs. > 30 years, *p* < 0.001; 20–30 years vs. > 30 years, *p* < 0.05). There was a statistically significant association between the graft used for the most recent revision ACL-R and the three age groups (*p* < 0.001).

**Table 1:** Demographic, surgical, and radiographic data of the total study group and the three pre-defined age groups

Variables	Total study group	Age group^a^			*p* value
		< 20 years	20–30 years	> 30 years	
Number of patients (*n*)	260	71	120	69	–
Age,^a^ (years)	26.2 ± 9.4 (13–58)	17.4 ± 1.5 (13–19)	23.9 ± 3.0 (20–30)	39.4 ± 6.9 (31–58)	< 0.001*
BMI, [kg/m^2^]	26.9 ± 5.0 (19.0–48.0)	25.4 ± 3.7 (20.0–40.0)	26.2 ± 4.8 (19.0–45.3)	29.8 ± 5.5 (21.2–48.0)	< 0.001*
Primary ACL-R to most recent revision ACL-R, [years]	6.1 ± 6.0 (0.2–40.0)	2.2 ± 1.6 (0.5–6.8)	5.5 ± 3.5 (0.2–14.8)	11.1 ± 8.6 (0.3–40.0)	< 0.001*
Males, *n* (%)	144 (55%)	34 (48%)	65 (54%)	45 (65%)	n.s
Right knee, *n* (%)	123 (47%)	35 (49%)	53 (44%)	35 (51%)	n.s
Number of revision ACL-R					n.s
First, *n* (%)	214 (82%)	66 (93%)	91 (76%)	57 (83%)	
Second, *n* (%)	35 (14%)	5 (7%)	21 (18%)	9 (13%)	
Third, *n* (%)	10 (4%)	0 (0%)	7 (6%)	3 (4%)	
Fourth, *n* (%)	1 (< 1%)	0 (0%)	1 (1%)	0 (0%)	
Graft^a^					< 0.001*
Hamstring, *n* (%)	8 (3%)	0 (0%)	3 (3%)	5 (7%)	
Quadriceps, *n* (%)	106 (41%)	34 (48%)	52 (43%)	20 (29%)	
BPTB, *n* (%)	88 (34%)	31 (44%)	42 (35%)	15 (22%)	
Allograft, *n* (%)	55 (21%)	6 (9%)	21 (18%)	28 (41%)	
Hamstring cont., *n* (%)	1 (< 1%)	0 (0%)	0 (0%)	1 (1%)	
BPTB cont., *n* (%)	2 (1%)	0 (0%)	2 (2%)	0 (0%)	
Graft diameter,^a^ [mm]	9.6 ± 0.7 (7.5–12.0)	9.7 ± 0.6 (8.0–11.0)	9.6 ± 0.7 (8.0–12.0)	9.4 ± 0.7 (7.5–11.0)	< 0.05*
Femoral graft fixation^a^					n.s
Suspensory, *n* (%)	179 (69%)	48 (68%)	83 (69%)	48 (70%)	
Interference, *n* (%)	38 (15%)	11 (16%)	16 (13%)	11 (16%)	
Over-the-top, *n* (%)	38 (15%)	12 (17%)	16 (13%)	10 (15%)	
Hybrid, *n* (%)	4 (2%)	0 (0%)	4 (3%)	0 (0%)	
N/A, *n* (%)	1 (< 1%)	0 (0%)	1 (1%)	0 (0%)	
Tibial graft fixation^a^					n.s
Suspensory, *n* (%)	66 (25%)	21 (30%)	29 (24%)	16 (23%)	
Interference, *n* (%)	160 (62%)	44 (62%)	71 (59%)	45 (65%)	
Hybrid, *n* (%)	32 (12%)	6 (9%)	18 (15%)	8 (12%)	
N/A, *n* (%)	2 (1%)	0 (0%)	2 (2%)	0 (0%)	
Contralateral ACL injury, *n* (%)	33 (13%)	12 (17%)	13 (11%)	8 (12%)	n.s
LFCR,^b^ [-]	0.65 ± 0.04 (0.52–0.84)	0.64 ± 0.05 (0.53–0.75)	0.65 ± 0.05 (0.55–0.84)	0.66 ± 0.04 (0.52–0.75)	n.s
Medial PTS,^b^ [°]	10.2 ± 3.3 (2.0–19.0)	9.9 ± 3.7 (4.0–19.0)	10.8 ± 3.3 (2.0–18.0)	9.4 ± 2.8 (3.2–15.0)	< 0.05*
Lateral PTS,^b^ [°]	9.5 ± 3.8 (1.0–22.0)	9.0 ± 4.3 (1.0–22.0)	10.3 ± 3.7 (3.0–20.0)	8.6 ± 3.4 (2.0–18.0)	< 0.05*

Categorical variables are expressed as mean (corresponding percentage). Continuous variables are expressed as mean ± standard deviation (range)

*ACL* anterior cruciate ligament, *ACL-R* ACL reconstruction, *BMI* body mass index, *BPTB* bone-patellar tendon-bone, *cont.* contralateral, *LFCR* lateral femoral condyle ratio, *N/A* not available, *n.s.* non-significant, *PTS* posterior tibial slope

*Statistically significant difference (*p* < 0.05)

^a^At most recent revision ACL-R

^b^Data available for 214 patients (82% of study group)

#### Intra-articular findings

A detailed overview is shown in Table 2. There were statistically significantly more patients with intact cartilage in the age group < 20 years compared to the other two age groups (*p* < 0.05). Patients age > 30 years had statistically significantly less intact cartilage at the medial femoral condyle (*p* < 0.001), lateral tibial plateau (*p* < 0.001), trochlea (*p* < 0.05), and patella (*p* < 0.001). Patients age > 30 years had also statistically significantly more high-grade cartilage lesions at the medial femoral condyle (*p* < 0.001) and patients age < 20 years had significantly more intact cartilage at the medial tibial plateau (*p* < 0.001).

**Table 2:** Intra-articular findings at most recent revision ACL-R of the total study group and the three pre-defined age groups

Variables	Total study group	Age group			*p* value
		< 20 years	20–30 years	> 30 years	
Meniscus lesion					n.s
None, *n* (%)	39 (15%)	12 (17%)	20 (17%)	7 (10%)	
Medial, *n* (%)	83 (32%)	17 (24%)	39 (33%)	27 (39%)	
Lateral, *n* (%)	48 (19%)	13 (18%)	18 (15%)	17 (25%)	
Both, *n* (%)	90 (35%)	29 (41%)	43 (36%)	18 (26%)	
Medial meniscal tear type					n.s
None, *n* (%)	87 (33%)	25 (35%)	38 (32%)	24 (35%)	
Horizontal, *n* (%)	2 (1%)	0 (0%)	1 (1%)	1 (1%)	
Longitudinal, *n* (%)	59 (23%)	19 (27%)	29 (24%)	11 (16%)	
Radial, *n* (%)	7 (3%)	0 (0%)	5 (4%)	2 (3%)	
Ramp, *n* (%)	21 (8%)	11 (16%)	5 (4%)	5 (7%)	
Complex, *n* (%)	31 (12%)	5 (7%)	17 (14%)	9 (13%)	
Root, *n* (%)	5 (2%)	0 (0%)	3 (3%)	2 (3%)	
s/p partial ME, *n* (%)	41 (16%)	8 (11%)	19 (16%)	14 (20%)	
s/p meniscal repair, *n* (%)	7 (3%)	3 (4%)	3 (3%)	1 (1%)	
Lateral meniscal tear type					n.s
None, *n* (%)	122 (47%)	29 (41%)	59 (49%)	34 (49%)	
Horizontal, *n* (%)	4 (2%)	1 (1%)	2 (2%)	1 (1%)	
Longitudinal, *n* (%)	30 (12%)	8 (11%)	14 (12%)	8 (12%)	
Radial, *n* (%)	11 (4%)	2 (3%)	4 (3%)	5 (7%)	
Complex, *n* (%)	11 (4%)	5 (7%)	4 (3%)	2 (3%)	
Root, *n* (%)	40 (15%)	16 (23%)	16 (13%)	8 (12%)	
s/p partial ME, *n* (%)	30 (12%)	8 (11%)	15 (13%)	7 (10%)	
s/p meniscal repair, *n* (%)	11 (4%)	2 (3%)	6 (5%)	3 (4%)	
N/A, *n* (%)	1 (< 1%)	0 (0%)	0 (0%)	1 (1%)	
Cartilage lesion, *n* (%)	190 (73%)	42 (59%)	91 (76%)	57 (83%)	< 0.05*
Cartilage medial femoral condyle					< 0.001*
Intact, *n* (%)	120 (46%)	42 (59%)	57 (48%)	21 (30%)	
Low-grade, *n* (%)	102 (39%)	23 (32%)	52 (43%)	27 (39%)	
High-grade, *n* (%)	38 (15%)	6 (9%)	11 (9%)	21 (30%)	
Cartilage medial tibial plateau					< 0.05*
Intact, *n* (%)	165 (63%)	57 (80%)	73 (61%)	35 (51%)	
Low-grade, *n* (%)	87 (34%)	14 (20%)	43 (36%)	30 (43%)	
High-grade, *n* (%)	8 (3%)	0 (0%)	4 (3%)	4 (6%)	
Cartilage lateral femoral condyle					n.s
Intact, *n* (%)	172 (66%)	48 (68%)	82 (68%)	42 (61%)	
Low-grade, *n* (%)	56 (22%)	15 (21%)	23 (19%)	18 (26%)	
High-grade, *n* (%)	32 (12%)	8 (3%)	15 (6%)	9 (4%)	
Cartilage lateral tibial plateau					< 0.05*
Intact, *n* (%)	181 (70%)	53 (75%)	91 (76%)	37 (54%)	
Low-grade, *n* (%)	62 (24%)	15 (21%)	23 (19%)	24 (35%)	
High-grade, *n* (%)	17 (6%)	3 (4%)	6 (5%)	8 (12%)	
Cartilage trochlea					< 0.05*
Intact, *n* (%)	214 (82%)	64 (90%)	101 (84%)	49 (71%)	
Low-grade, *n* (%)	27 (10%)	7 (10%)	9 (8%)	11 (16%)	
High-grade, *n* (%)	19 (7%)	0 (0%)	10 (8%)	9 (13%)	
Cartilage patella					< 0.05*
Intact, *n* (%)	199 (77%)	60 (85%)	97 (81%)	42 (61%)	
Low-grade, *n* (%)	54 (21%)	11 (15%)	21 (18%)	22 (32%)	
High-grade, *n* (%)	7 (3%)	0 (0%)	2 (2%)	5 (7%)	

Categorical variables are expressed as mean (corresponding percentage)

*ACL-R* anterior cruciate ligament reconstruction, *ME* meniscectomy, *N/A* not available, *n.s.* non-significant, *s/p* status post

*Statistically significant difference (*p* < 0.05)

#### Concurrent procedures

A detailed overview is shown in Table 3. Statistically significantly more medial meniscal repairs were performed in the age group < 20 years (*p* < 0.05). Concurrent osteotomies were performed significantly more often in the age group > 30 years (23%, *p* < 0.05). In patients with concurrent LET (31%), significantly more intact cartilage was observed at the medial femoral condyle (64% vs. 38%, *p* < 0.001), lateral femoral condyle (78% vs. 61%, *p* < 0.05), medial tibial plateau (80% vs. 56%, *p* < 0.001), lateral tibial plateau (84% vs. 63%, *p* < 0.05), and at the trochlea (94% vs. 77%, *p* < 0.05) compared to patients without concurrent LET. In addition, preoperative Lachman and pivot-shift tests were significantly more frequently classified as severely abnormal in patients with than without concurrent LET (Lachman, 42% vs. 21%, *p* < 0.05; pivot-shift, 42% vs. 19%, *p* < 0.001).

**Table 3:** Concurrent procedures at most recent revision ACL-R of the total study group and the three pre-defined age groups

Variables	Total study group	Age group			*p* value
		< 20 years	20–30 years	> 30 years	
Medial meniscus treatment					< 0.05*
No treatment, *n* (%)	117 (45%)	31 (44%)	52 (43%)	34 (49%)	
Repair, *n* (%)	68 (26%)	28 (39%)	25 (21%)	15 (22%)	
Partial ME, *n* (%)	51 (20%)	9 (13%)	29 (24%)	13 (19%)	
MAT, *n* (%)	24 (9%)	3 (4%)	14 (12%)	7 (10%)	
Lateral meniscus treatment					n.s
No treatment, *n* (%)	170 (65%)	43 (61%)	80 (67%)	47 (68%)	
Repair, *n* (%)	49 (19%)	16 (23%)	21 (18%)	12 (17%)	
Partial ME, *n* (%)	37 (14%)	11 (16%)	16 (13%)	10 (15%)	
MAT, *n* (%)	4 (2%)	1 (1%)	3 (3%)	0 (0%)	
PCL treatment					n.s
None, *n* (%)	258 (99%)	70 (99%)	120 (100%)	68 (99%)	
Repair, *n* (%)	0 (0%)	0 (0%)	0 (0%)	0 (0%)	
Reconstruction, *n* (%)	2 (1%)	1 (1%)	0 (0%)	1 (1%)	
MCL treatment					n.s
None, *n* (%)	253 (97%)	71 (100%)	117 (98%)	65 (94%)	
Repair, *n* (%)	0 (0%)	0 (0%)	0 (0%)	0 (0%)	
Reconstruction, *n* (%)	7 (3%)	0 (0%)	3 (3%)	4 (6%)	
LCL treatment					n.s
None, *n* (%)	259 (100%)	70 (99%)	120 (100%)	69 (100%)	
Repair, *n* (%)	0 (0%)	0 (0%)	0 (0%)	0 (0%)	
Reconstruction, *n* (%)	1 (< 1%)	1 (1%)	0 (0%)	0 (0%)	
Osteotomy					< 0.05*
None, *n* (%)	227 (87%)	67 (94%)	107 (89%)	53 (77%)	
HTO slope reducing	13 (5%)	3 (4%)	6 (5%)	4 (6%)	
HTO-MOW, *n* (%)	13 (5%)	0 (0%)	3 (3%)	10 (15%)	
HTO-LCW, *n* (%)	3 (1%)	0 (0%)	2 (2%)	1 (1%)	
DFO-MCW, *n* (%)	3 (1%)	1 (1%)	1 (1%)	1 (1%)	
HTO-MCW, *n* (%)	1 (< 1%)	0 (0%)	1 (1%)	0 (0%)	
Lateral extra-articular tenodesis, *n* (%)	81 (31%)	25 (35%)	41 (34%)	15 (22%)	n.s
Cartilage surgery					n.s
None, *n* (%)	237 (91%)	66 (93%)	107 (89%)	64 (93%)	
OATS autograft, *n* (%)	5 (2%)	2 (3%)	2 (2%)	1 (1%)	
OATS allograft, *n* (%)	2 (1%)	0 (0%)	2 (2%)	0 (0%)	
Microfracture, *n* (%)	14 (5%)	3 (4%)	8 (7%)	3 (4%)	
MACI, *n* (%)	1 (< 1%)	0 (0%)	1 (1%)	0 (0%)	
PJAC, *n* (%)	1 (< 1%)	0 (0%)	0 (0%)	1 (1%)	

Categorical variables are expressed as mean (corresponding percentage)

*ACL-R* anterior cruciate ligament reconstruction, *DFO* distal femoral osteotomy, *HTO* high tibial osteotomy, *LCL* lateral collateral ligament, *LCW* lateral closed wedge, *MACI* matrix-induced autologous chondrocyte implantation, *MAT* meniscal allograft transplantation, *MCL* medial collateral ligament, *MCW* medial closed wedge, *ME* meniscectomy, *MOW* medial open wedge, *n.s.* non-significant, *OATS* osteochondral transplantation, *PCL* posterior cruciate ligament, *PJAC* particulated juvenile articular cartilage

*Statistically significant difference (*p* < 0.05)

### Trends over time

**Fig. 2:**
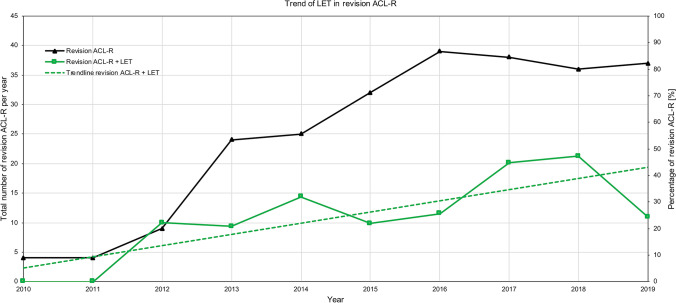
Trend of combined revision ACL-R + LET. The black line represents the total number of revision ACL-Rs performed annually (left scale). The green line represents combined revision ACL-Rs + LET as a percentage of the total number of revision ACL-Rs performed annually (right scale). The dotted green line represents the trendline of combined revision ACL-Rs + LET, indicating a statistically significant linear increase over time (*p* < 0.05; adjusted *R*^2^ = 0.658). *ACL-R* anterior cruciate ligament reconstruction, *LET* lateral extra-articular tenodesis

Revision ACL-R was performed in 66 patients (58% male) with a mean age of 25.7 ± 8.1 years (range 15–48 years) between 2010 and 2014 and in 194 patients (55% male) with a mean age of 26.4 ± 9.8 years (range 13–58 years) between 2015 and 2020. Quadriceps tendon autograft was used significantly more often in 2015–2020 compared to 2010–2014 (49% vs. 18%, *p* < 0.001). More medial [27% vs. 23%, non-significant (n.s.)] and lateral meniscal repairs (21% vs. 14%, n.s.), osteotomies (13% vs. 11%, n.s.), and LETs (34% vs. 23%, n.s.) were performed in 2015–2020 compared to 2010–2014. Time series analysis revealed a statistically significant and positive linear relationship between the time period (2010–2020) and the percentage of combined revision ACL-R + LET among revision ACL-R performed annually (*p* < 0.05; adjusted *R*^2^ = 0.658; Fig. 2). No statistically significant relationship could be observed between the proportion of other concurrent procedures (meniscal repair, meniscectomy, MAT, osteotomy) among the revision ACL-Rs performed annually.

## Discussion

The most important finding of this study was that quadriceps tendon autografts and concurrent LET are becoming increasingly popular in revision ACL-R, especially in younger patients. Young patient age was also associated with less time between primary ACL-R and the most recent revision ACL-R, more medial meniscal repairs, and higher rates of intact cartilage. In addition, a high and growing rate of concurrent meniscal repairs (45%), LET (31%), and osteotomies (13%) was observed, underscoring the high surgical demands of revision ACL-R. Moreover, LET in revision ACL-R was associated with intact cartilage and severely abnormal preoperative knee laxity.

Graft choice and availability in the setting of revision ACL-R are different than for primary ACL-R, especially if multiple graft failures have already occurred. Previous research highlighted the importance of graft choice in the setting of revision ACL-R, as it was demonstrated that graft choice represents a significant predictor for functional outcomes and graft failure rates at 2-year follow-up [24]. Between 2006 and 2011, 1205 patients undergoing revision ACL-R were enrolled in the MARS cohort. Allografts were used in 49%, autografts in 48%, and a combination of allografts and autografts in 3% for revision ACL-R. Regression analyses revealed that the use of autografts in the setting of revision ACL-R resulted in improved patient reported outcomes, sports function, and a significantly decreased risk of subsequent graft failure compared to the use of allografts [24]. The clinical impact of previous research is reflected in the findings of this study, where a notable decrease in the use of allografts (21%) and an increase in the use of autografts (79%) compared to the MARS cohort was observed. Looking at European countries, where allografts are often not readily available, the different types of autografts used for revision ACL-R can be evaluated [23]. The Danish Registry for Knee Ligament Reconstructions, the Norwegian Knee Ligament Registry, and the revision ACL-R cohort of the Société Française d’Arthroscopie showed that the most frequently used types of autografts for revision ACL-R were hamstring tendon (39–56%), followed by bone-patellar tendon-bone (28–56%), and quadriceps tendon (2%) [22, 23]. In this study, hamstring tendon (ipsilateral + contralateral), bone-patellar tendon-bone (ipsilateral + contralateral), and quadriceps tendon autografts were used in 4%, 35%, and 41% of patients, respectively. While the percentage of bone-patellar tendon-bone autografts used is consistent with previous reports, in this study, hamstring and quadriceps tendon autografts are strikingly under- and over-represented, respectively. An almost eightfold increase in the use of quadriceps tendon autograft was observed in the second (2015–2020) compared to the first (2010–2014) half of the observation period of this study. Accordingly, quadriceps tendon autograft represents the currently most frequently used graft for revision ACL-R in the two participating centers. The reason for the shift in autograft types used for revision ACL-R may be due to the emerging evidence of increased failure rates for hamstring tendon autografts and decreased failure rates for quadriceps tendon autografts in ACL-R [12, 38, 46, 49].

Failures in ACL-R may also be caused by persistent rotatory knee laxity. Improved anatomical and biomechanical knowledge of the antero-lateral structures of the knee has increased the awareness of antero-lateral rotatory knee laxity as a cause of ACL graft failure [11, 18, 19]. A randomized controlled trial has demonstrated statistically significantly less ACL graft failures in patients undergoing primary ACL-R + LET (4%) compared to patients undergoing isolated hamstring tendon autograft primary ACL-R (11%) [12]. Revision ACL-R is often considered the primary indication for an additional LET, with good mid-term outcomes and low failure rates reported [1, 16, 48]. In this study, 31% of patients underwent concurrent LET, with the majority of patients receiving LET being < 30 years old. Patients undergoing concurrent LET were characterized by severely abnormal preoperative knee laxity and low cartilage wear.

Numerous studies have demonstrated the chondroprotective and stabilizing role of the menisci, underscoring their vital role in maintaining normal knee kinematics and function [3, 20, 40]. As a result, meniscal repairs are increasing and high success rates have been reported in the setting of revision ACL-R [26, 43]. In one study, 18% of patients undergoing revision ACL-R underwent concurrent meniscal repair, with more than two-thirds of repairs accounting for the medial meniscus and an overall failure rate of meniscal repairs of 9% [26]. Similarly, in the current study, more medial than lateral meniscal repairs were performed (26% vs. 19%), with a total of 45% of patients undergoing concurrent meniscal repair. Patients < 20 years (39%) were significantly more likely to undergo concurrent medial meniscal repair than patients aged 20–30 years (21%) or > 30 years (22%).

Certain bony morphological characteristics of the proximal tibia and the distal femur have been associated with an increased risk for primary and recurrent ACL injuries [14, 15, 41, 45]. The PTS, as a surgically modifiable risk factor for ACL injuries, has attracted special attention in recent years. Clinical observations demonstrated a positive correlation between PTS and anterior tibial subluxation and rotatory knee laxity [14, 47]. The mechanical impact of the PTS on ACL grafts has also been confirmed by several biomechanical studies, demonstrating increased ACL graft forces with increased PTS [4, 50]. Therefore, slope-reducing high tibial osteotomies have been proposed to counteract the negative effects of increased PTS on ACL grafts [9, 21, 51]. In this study, 13% of patients underwent concurrent osteotomies, with 40% of osteotomies representing slope-reducing osteotomies, indicating an increased awareness of the PTS as a potential cause of ACL graft failure. Consistent with previous reports, it was also shown that the LFCR is significantly higher in female compared to male patients [45], which may also be the reason for the significantly higher rate of contralateral ACL injuries in female patients.

Revision ACL-R has been shown to result in inferior patient reported outcomes (4–8 points less in Lysholm Score; 5–19 points less in Knee Injury and Osteoarthritis Outcome Score subscales) and increased residual laxity compared to primary ACL-R [13, 17, 37, 42, 53, 55, 56]. Independent modifiable predictors for inferior outcomes and higher failure rates after revision ACL-R compared to primary ACL-R have been identified and include graft choice and persistent rotatory knee laxity caused by increased PTS, loss of meniscal tissue, and peripheral capsuloligamentous insufficiency. This study demonstrated increased awareness of modifiable risk factors in revision ACL-R by an increasing number of concurrently performed procedures to address independent predictors of worse outcomes and thus reduce the risk of subsequent ACL graft failures. Future studies should elaborate on specific indications for concurrent surgical procedures to facilitate decision-making in revision ACL-R.

The retrospective nature of this study is associated with several limitations. Since most of the patients had their previous ACL-Rs performed in a hospital other than one of the two participating centers, it was not possible to report changes of intra-articular findings from primary ACL-R to most recent revision ACL-R. However, the main objective of this study was to evaluate trends in revision ACL-R, which was possible due to the large sample size. Including two centers and three experienced knee surgeons reduced selection bias and thus increases the generalizability of the presented data. Despite the observation of increasing complexity in revision ACL-R compared to previous reports, it is currently unknown how this affects clinical and functional outcomes, as patient reported outcomes were not collected in this study.

## Conclusions

This study showed that quadriceps tendon autograft is becoming increasingly popular in revision ACL-R, especially in younger patients. In addition, a high and growing rate of concurrent meniscal repairs (45%), LET (31%), and osteotomies (13%) was observed, underscoring the high surgical demands of revision ACL-R. Lateral extra-articular tenodesis was associated with intact cartilage and severely abnormal preoperative knee laxity, which represent indications for LET in revision ACL-R.
